# Acute effects of a short bout of walking on domain-specific cognition in adults with knee replacement

**DOI:** 10.1093/geroni/igaf122.3223

**Published:** 2025-12-31

**Authors:** Jongwon Lee, Scott Jamieson, Clare Kennerley, Christine Pellegrini, Chih-Hsiang Yang

**Affiliations:** University of South Carolina, Columbia, South Carolina, United States; University of South Carolina, Columbia, South Carolina, United States; University of South Carolina, Columbia, South Carolina, United States; University of South Carolina, Columbia, South Carolina, United States; University of South Carolina, Columbia, South Carolina, United States

## Abstract

The total knee replacement (TKR) is a common elective surgery in older adults, and adults with TKR remain physically inactive and are at high risk for Alzheimer’s and related dementia (ADRD). Shorter bouts of walking may be more feasible for this population to reduce the risk of ADRD because prolonged physical activity could be challenging due to lower physical functioning. This study investigated the acute effects of the 6-minute walk on processing speed, visuospatial memory, and visual memory in adults with a history of TKR ≤ 6 months ago. They were randomized into two sequences with two periods: 6 minutes of walking or resting first. Cognition was assessed via brief phone-based tests at baseline, immediately after the first and second periods. Symbol search, grid memory, and color shapes tests were used to measure processing speed, visuospatial memory, and visual memory, respectively. The analysis included 52 participants (female = 40 (77%), age = 65.06 (9.06) yrs, time since surgery = 94.08 (21.66) days). Controlling for demographics and potential learning effects, mixed-effects models revealed that 6 minutes of walking was associated with improved visuospatial memory, as indicated by shorter error distances on the grid memory test (b = -3.87, p = 0.04). The walking effect was not observed for processing speed (b = 178.82, p = 0.45) or visual memory (b = 0.01, p = 0.92). Although the physiological mechanisms to which exercise benefits domain-specific cognition remain unclear, shorter bouts of walking may immediately improve visuospatial memory in this population.

